# Cellular Phone Enabled Non-Invasive Tissue Classifier

**DOI:** 10.1371/journal.pone.0005178

**Published:** 2009-04-13

**Authors:** Shlomi Laufer, Boris Rubinsky

**Affiliations:** Center for Bioengineering in the Service of Humanity and Society, School of Computer Science and Engineering, Hebrew University of Jerusalem, Jerusalem, Israel; The University of Queensland, Australia

## Abstract

Cellular phone technology is emerging as an important tool in the effort to provide advanced medical care to the majority of the world population currently without access to such care. In this study, we show that non-invasive electrical measurements and the use of classifier software can be combined with cellular phone technology to produce inexpensive tissue characterization. This concept was demonstrated by the use of a Support Vector Machine (SVM) classifier to distinguish through the cellular phone between heart and kidney tissue via the non-invasive multi-frequency electrical measurements acquired around the tissues. After the measurements were performed at a remote site, the raw data were transmitted through the cellular phone to a central computational site and the classifier was applied to the raw data. The results of the tissue analysis were returned to the remote data measurement site. The classifiers correctly determined the tissue type with a specificity of over 90%. When used for the detection of malignant tumors, classifiers can be designed to produce false positives in order to ensure that no tumors will be missed. This mode of operation has applications in remote non-invasive tissue diagnostics in situ in the body, in combination with medical imaging, as well as in remote diagnostics of biopsy samples in vitro.

## Introduction

Telemedicine, the use of telecommunication in medicine, is becoming an increasingly important branch of medicine. A recent review of different wireless and networking technologies and their use to promote the ultimate goal of global health by means of deployment of a telemedicine paradigm is found in [1]. Telemedicine finds applications in almost every aspect of medicine. For instance, sampling signals from sensors on patients and transmits digital data over a Bluetooth link to a mobile telephone was discussed in [2]. The use of wireless technologies, such as wireless LAN and sensor networks, for remote cardiac patient monitoring (telecardiology) was discussed in [3]. A comprehensive 3G universal mobile telecommunications system (UMTS) solution for the delivery of voice, real-time video, ECG signals, and medical scans information from an ambulance to a hospital was presented in [4].The use of medical imaging through a telecommunication network for minimally invasive surgery was introduced in 2004 [5], [6].

Recently, our group has expanded on our previous work in telemedicine [5], [6] and introduced the design of a conceptually new device technology in which the data processor site and the data acquisition site are geographically separated, and a cellular phone is used as a conduit of raw and processed data between the two distant sites [7]. One possible application of this technology is for the majority of the world population currently without access to medical imaging [7]. In another recent paper [8], we introduced the use of classifier technology for tissue characterization in X-ray mammography. Conventional mammography can identify areas of suspicious tissue, and often invasive needle biopsy is used to determine if the suspicious tissue is benign or malignant. We proposed a new way to characterize the suspicious tissue without the use of needle biopsies. The method works by combining knowledge of the location and size of the tissue sample of interest from X-ray mammography with multi-frequency electrical measurements made on the breast surface (in a configuration similar to the mammogram, as in [9]) and with Support Vector Machine (SVM) classifier techniques. The classifying capability is due to the fact that malignant tumors have different electrical properties from benign tumors [9]–[14]. The study was theoretical and demonstrated the feasibility of the concept.

In this study, we combine the cellular phone technology of [7] with the classifier technique of [8] for a new method of tissue characterization through the cellular phone. This study has experimental and theoretical aspects. The main aim of the study is to produce the first experimental demonstration of the feasibility of the theoretical concept introduced in [8]. The experimental study was done with tissue samples *in vitro*. This particular experimental technique may also have immediate use for the characterization of tissues from biopsies. Tissue biopsies are a standard diagnostic tool. In major hospitals, experts in histology can perform the tissue analysis on site, immediately after the biopsies are taken. However, biopsy samples taken at smaller clinics and by private physicians are usually sent out for histological analysis. This is a lengthy process which inconveniences patients and increases the cost of the treatment. The technique shown in this study could be used for biopsy tissue characterization at the site where it is taken, from a distance through the cellular phone.

This paper is presented in two parts. In the first, we introduce the experimental procedure, the data acquisition device (DAD) electrical hardware, and the classifier software and demonstrate how we train the classifier to distinguish between tissue types. In the second part, we introduce the use of the cellular phone to connect between the DAD at a remote data sampling site and the trained classifier at a central location.

## Materials and Methods

### Biological Samples and Electrical Measurements

All procedures complied with the National Institute of Health Guide for the care and use of Laboratory Animals and were approved by the Institutional Animal Care and Use Committee of the Hebrew University, Jerusalem, Israel.

### Animal procedures

Male Sprague-Dawley rats, weighing 250–300 grams, were obtained from Harlan Laboratories in Jerusalem, Israel. On the day of the procedure, the rats were anesthetized using a ketamine/xylasine combination (40 mg/kg and 10 mg/kg, respectively). Fifteen hearts and 24 kidneys were taken from 15 different rats. (To minimize animal use, the organs were taken from animals that were used for other studies immediately after the animals were sacrificed). The average weights of the organs are listed in Table 1. It should be noted that the heart and kidney tissues had similar weights. After removal, the fresh organs were placed in saline with heparin and refrigerated at 10°C until the measurements were performed. All the measurements were done within three hours of each rat's sacrifice, and the order of the measurements followed the order in which the animals were sacrificed. However, the order in which the measurements were done for the different organs was random.

**Table 1:** Average weight.

	Mean	Std
Rat	281.47 gr.	8.76
Kidney	1.65 gr.	0.153
Heart	1.42 gr.	0.149

**Figure 1:**
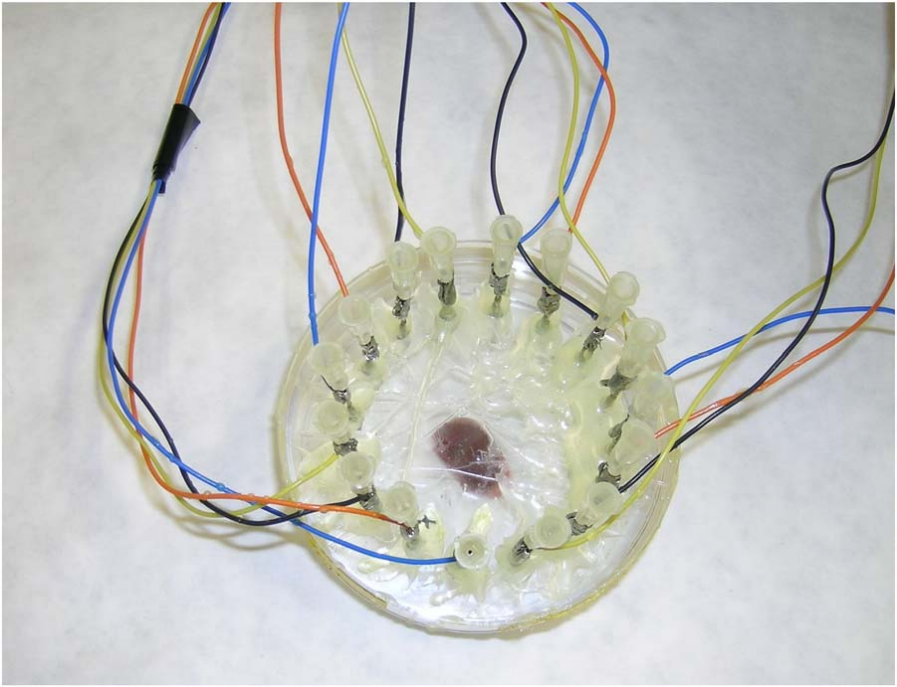
Measurement configuration. The kidney is placed in the center of a 9 cm-diameter Petri dish and covered with 25 ml of saline. Sixteen holes, equally distributed on a 6 cm-diameter circle, were made in the cover of the Petri dish. The electrodes were placed through these holes.

**Figure 2:**
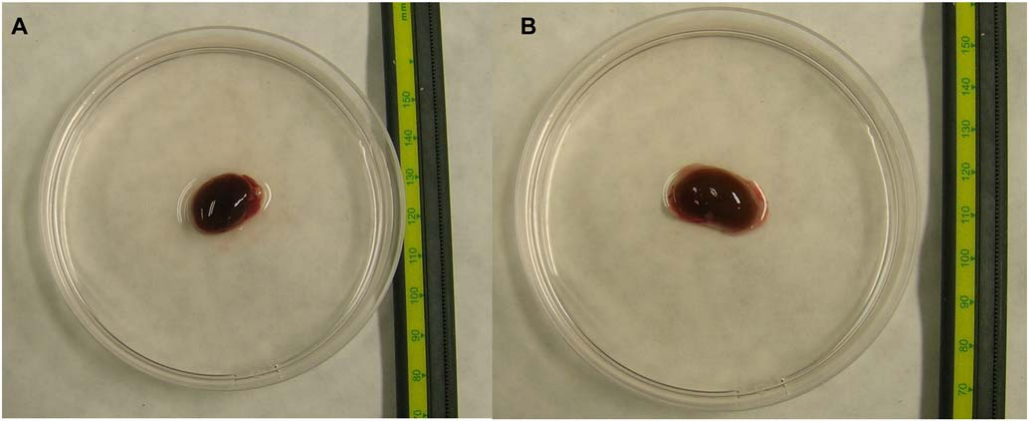
Organ placement. a) Heart; and b) Kidney.

The measurement device is shown in Figure 1. Sixteen holes, equally distributed on a 6 cm-diameter circle, were made in the cover of a 9 cm-diameter Petri dish. Needles (1.10×38 mm) were inserted through the holes, as exhibited in Figure 1. For each measurement, the organ was positioned in the center of the 9 cm-diameter Petri dish in 25 ml of saline and covered as described above. An image of the organ in the Petri dish can be seen in Figure 2.

**Figure 3:**
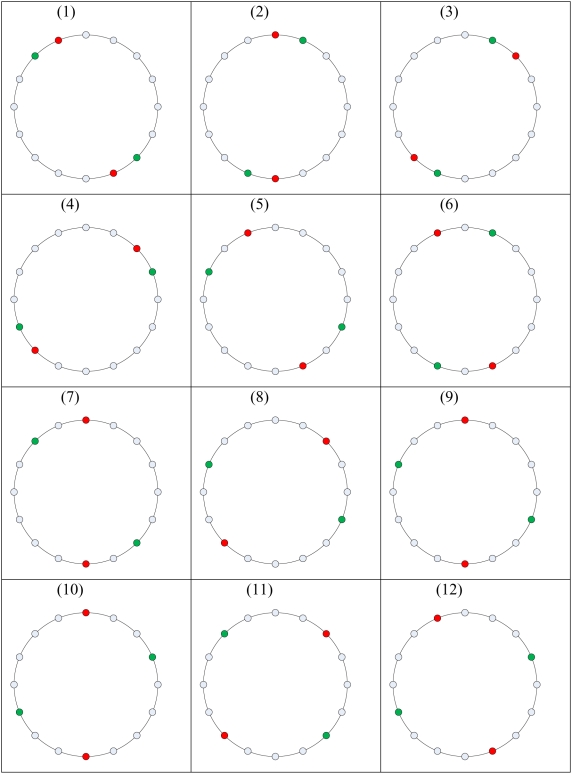
Electrode combinations. Red electrodes are used for current injunction, and green electrodes are used for voltage measurement. The combinations can be divided into four groups: I) Combinations 1–4, adjacent electrodes; II) Combinations 5–7, only one electrode away; III) Combinations 8–10, two electrodes away; and IV) Combinations 11–12, three electrodes away.

All impedance measurements were performed using a custom-developed impedance analyzer embedded in a single Printed Circuit Board (PCB). The impedance analyzer architecture is described in [15]. A total of 11 different frequencies, ranging from 1 kHz to 400 kHz, were measured. Using a manual switchboard, 12 different electrode configurations were employed. In each configuration, four electrodes were used, with two opposite electrodes for current injection and two opposite electrodes for voltage measurement. The different configurations are depicted in Figure 3. The data from each configuration were collected for five seconds.

### Support Vector Machines

Support Vector Machines are a group of supervised learning methods belonging to the family of generalized linear classifiers [16], [17]. The fundamental principle is to map the input vectors, *x*, into a high-dimensional feature space, *z*, through some nonlinear mapping chosen *a priori*. An optimal separating hyperplane is then constructed in this space as a method for characterization [18]. SVMs have numerous uses, ranging from general applications, such as object recognition [19], speaker identification [20], face detection [21], and text categorization [22], to applications that are more relevant to this study, such as mammogram classification [23], [24].

For the convenience of readers in life sciences, we state here the classical formulation of a classifier problem. A more intuitive explanation can be found in Appendix S1, and a more complete tutorial can be found in [16], [17], as well as in machine learning textbooks, such as [25]. Briefly, the problem solved using SVMs is as follows: We want to separate the set of training vectors belonging to two different classes:
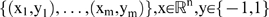
(1)


Using the hyperplane:
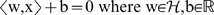
(2)


The decision function corresponding to this is:
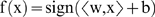
(3)


This leads us to the minimization problem:
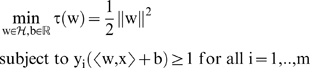
(4)


In order to solve this problem, we can use Lagrange multipliers α_i_≥0 and a Lagrangian
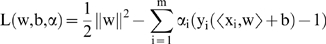
(5)


L has to be: a) minimized with respect to the primal variables *w*, and b) maximized with respect to the dual variables α_i_ (in other words, we are looking for a saddle point). The vectors x_i_ for which α_i_≠0 will be called support vectors.

Since the derivatives of L with respect to the primal variables must vanish, we get:
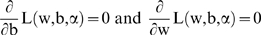
(6)


Leading to:
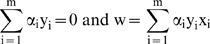
(7)


The dual problem will take the form:
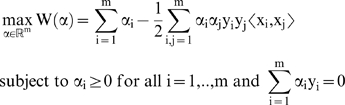
(8)


We will use a kernel k(x,x′) in order to map from the input space to a feature space.

Since a separating hyperplane may not exist, we will relax the constraints:
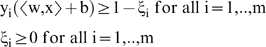
(9)


The objective function will change too:
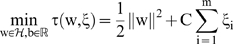
(10)


Leading us to our final optimization problem of:
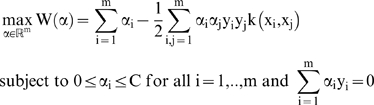
(11)


And our decision function will be:
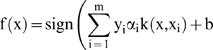
(12)


In the present case, each entry in the vector 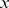 is a voltage value (amplitude or phase) measured for a different electrical excitation frequency. Since 11 frequencies were used, then n = 11. 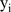 corresponds to the tissue type, 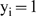 for a kidney and 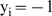 for a heart. The index: 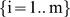 is one index for each different organ, meaning 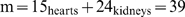.

### SVM Classifier Training

The SVM was trained using svmLight [26]. Two types of inputs were examined: the voltage *magnitude* and the voltage *phase*. The magnitude data were normalized in the following way:
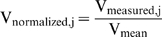
(13)


Where 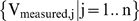 are a set of measurements (n frequencies) for a specific electrode configuration and 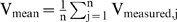. The phase data were not changed.

It should be noted that the tissue samples had a similar geometry and shape and were placed in similar locations relative to the measurement electrodes. This information is part of the information on which the classifier operates. The equivalent of this information for *in vivo* use of classifiers are the size and location of the unknown tissues in the body relative to the measurement electrodes, which in clinical practice will be obtained from mammography or other imaging techniques (see [8]). As mentioned earlier, 12 different electrode combinations were examined. Given that only opposite electrodes were used, the different combinations can be divided into four groups according to the distance between the electrodes, namely adjacent electrodes and electrodes separated by one, two, or three electrodes, (see Figure 3).

In the first stage, classifiers were trained for all of the combinations. Then each classifier was given a binary score (1 for kidney and −1 for heart), and the classifiers were summed. Since better classification results were obtained from the configurations where the current electrodes were close to the voltage electrodes, the final classifier used only two groups: adjacent electrodes (configurations 1–4 in Figure 3) and electrodes separated by only one other electrode (configurations 5–7 in Figure 3). Thus, the final classifier was constructed from seven electrode configurations. This method of summing several classifiers of different electrode combinations has shown better stability than using just one electrode combination.

### Cellular Phone Technology

The hardware (data acquisition device – DAD) was separated from the computer that processed the data for classification. The DAD was connected to a low-end computer, which only averaged and saved the electrical data measurements and then sent them through Bluetooth to the cellular phone. We used a low-end computer because of subjective cost considerations. However, in a real application of this technology, the board that makes the DAD can be upgraded, relatively inexpensively, in order to connect directly to the cellular phone using USB or Bluetooth. Therefore, a real application would not necessitate a computer at the patient site. The cellular phone used was a Nokia N95, and the local cellular phone service used was Orange TM.

**Figure 4:**
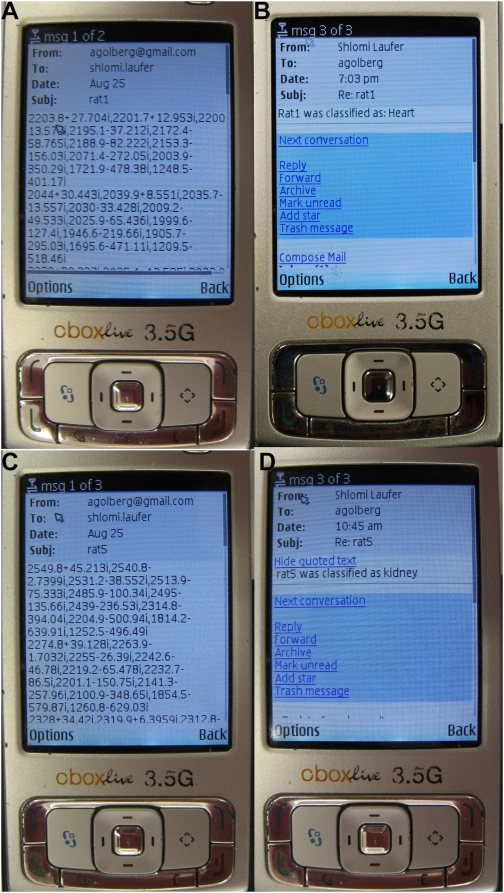
Implementation of classifier using the cellular phone. a) Measured rat heart data sent; b) Heart result received; c) Measured rat kidney data sent; and d) Kidney result received.

In order to test the cellular phone concept, the data were arranged in a text file containing about 1200 bytes and sent via e-mail from the cellular phone to the remote computer. Each File had seven lines, one for each different electrode configuration. Each line consisted of eleven complex numbers, one for each frequency. Once the file was received on the remote computer (AMD Athlon 64 X2 Dual Core Processor 5000+, 2.61 GHz, 2 GB RAM, Microsoft Windows XP), the SVM classifier program was applied to the data and a classifier score was calculated. Accordingly, the tissue was classified as either kidney or heart. The remote computer then sent an email reply to the DAD site with the word heart or the word kidney. The process is demonstrated in Figure 4.

## Results

**Figure 5:**
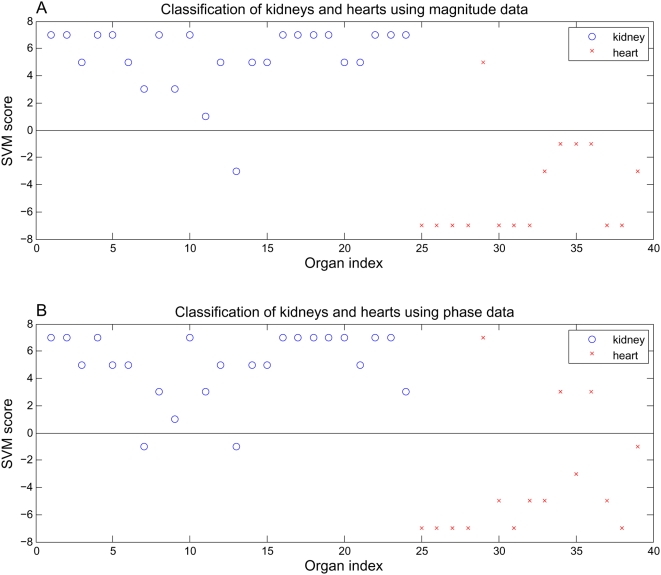
Classification of kidneys and hearts. a) The SVM score of the final classifier, using the magnitude data; and b) the SVM score of the final classifier, using the phase data.

Separate classifiers were trained for all electrode combinations, using the voltage amplitude data and the phase data, with the method developed in [8]. The training error was calculated using the leave-one-out cross-validation method [27]. The results summary can be seen in Table 2. The table presents the results for 12 different classifiers that operated using the voltage magnitude data of the 12 electrode configurations, as well as 12 classifiers that operated using the voltage phase data. It also includes the results of two final classifiers that were constructed using a combination of the previous classifiers, one from the magnitude data and one from the phase data. For the construction of the final classifier, seven electrode configurations were used. They were each given a binary score (−1, 1) and were summed to obtain the final two classifiers. The SVM final score can be seen in Figure 5.

**Table 2:** Classifier results.

Electrode Combination	Magnitude Data		Phase Data	
	Sensitivity	Specificity	Sensitivity	Specificity
1	83.3%	73.3%	83.3%	80%
2	95.8%	86.7%	91.7%	73.3%
3	79.1%	80%	87.5%	73%
4	91.6%	86.7%	91.7%	80%
5	87.5%	86.7%	87.5%	73.3%
6	91.6%	80%	66.7%	80%
7	91.6%	80%	91.7%	66.7%
8	83.3%	73.3%	87.5%	86.7%
9	83.3%	60%	79.2%	53.3%
10	83.3%	66.7%	83.3%	93.3%
11	66.7%	60%	50%	33.3%
12	58.3%	73.3%	62.5%	66.7%
Final Classifier	95.8%	93.3%	91.7%	80%

The electrode combinations refer to the combinations shown in Figure 3. The final classifier is the majority score of the first seven single electrode combination classifiers. Combinations 1–4 are for adjacent electrodes, and combinations 5–7 are for electrodes separated by only one other electrode.

## Discussion

Classifiers were trained for all 12 electrode configurations so as to be divided into four groups of configurations in accordance with the distances between the electrodes. It can be seen in Table 2 that the configurations in which the current electrodes and voltage electrodes were closer to each other gave better results. Since the classifier results for electrodes that are farther away from each other are still above the random score of 50%, they can be used in order to improve the final classifier. It can also be seen in Table 2 that although configurations 1–4 and configurations 5–7 are respectively symmetrical, there are differences in the classifier results. The combination of several measurements for the final classifier is more stable to noise and produces better results.

More complex methods can be employed, such as giving different weights to different configurations. For example, closer electrodes (i.e., configurations 1–4) can be given higher weights than farther electrodes (i.e., configurations 8–10). This may allow the utilization of more electrodes, such as configurations 8–10, which failed to improve the results and thus were not included in the final classifier. However, for purposes of this preliminary study, using the majority method seemed sufficient.

Two types of datasets were used for the ex-vivo biological model classifier, one using the amplitude data and the other using the phase data. It can be seen that both classifiers gave about 90% correct classification. We have not tried to optimize the classifier developed here for any particular performance. However, it should be possible to design a classifier that can be biased to produce only false positive so as not to miss malignant tissue at the expense of a higher level of false positive result.

The classifier based on amplitude measurements yielded slightly better results than that based on phase data, but the database is too small to reach any final conclusions. The potential advantage in the phase data is its inherent normalization [28], rendering it less subject to influence by tumor size, which was less of an issue in this study. Furthermore, it has been shown that for some tissue types, the main discriminator will be the phase data rather than the magnitude [29].

The study presented here has demonstrated the feasibility of the classifier concept for tissue identification using heart and kidney tissue. These tissues were chosen to demonstrate the concept because they were available from another study and our research strategy is to minimize the use of animals in research. However, the concept developed in this study is general and is relevant to distinguishing between any types of tissue with different electrical properties in a range of frequencies. To illustrate this point we bring here tables 3 and 4. The tables give the electromagnetic properties of heart and kidney tissue as a function of frequency [30], [31]. In addition the table gives the ratio between the properties of the heart and kidney at the same frequency. Obviously the greater the difference between two tissue properties the easier it is to distinguish between them. The tables also give the electromagnetic properties of breast carcinoma and breast fibroadenoma [11], [12]. An important use of this technique would be to distinguish between a benign and malignant breast tumor from biopsies. The table shows that the ratio between the electromagnetic properties of these two types of breast tumors is significant and even larger than between heart and kidney. This suggests that the classifier technique introduced here could be used to distinguish between tissues in clinical situations of importance

**Table 3:** Magnitude of impedance (given in Ωcm). CA(Carcinoma) - malignant tumors; FA (Fibroadenoma) - benign tumors of the breast.

Frequency(Hz)	977	1950	3910	7810	15630	31250	62500	1.25E+05	2.50E+05
CA	369	363	357	350	342	331	319	305	290
FA	244	242	239	235	231	225	221	216	212
Ratio	1.51	1.5	1.49	1.49	1.48	1.47	1.44	1.41	1.37
Heart	927.47	841.03	740.95	654.79	586.7	531.07	482.01	435.68	388.82
Kidney	883.24	841	786.98	734.54	687.88	644.74	600.33	548.47	482.56
Ratio	1.05	1.00	0.94	0.89	0.85	0.82	0.80	0.79	0.81

Values for breast taken from [12], definitions taken from [11]. The values for kidney and heart were computed using the model and values in [30], also described in [31].

**Table 4:** Phase of impedance.

Frequency(Hz)	977	1950	3910	7810	15630	31250	62500	1.25E+05	2.50E+05
CA	1.5	2.1	2.7	3.3	4	4.8	6.1	8	10.1
FA	0.7	1.2	1.8	2.5	2.8	3.3	3.5	4.3	4.9
Ratio	2.14	1.75	1.5	1.32	1.42	1.45	1.74	1.86	2.06
Heart	−10.36	−13.19	−14.54	−14.3	−13.7	−13.4	−13.74	−14.65	−16.21
Kidney	−5.95	−7.34	−8.33	−8.77	−9.19	−10.1	−12	−15.07	−19.12
Ratio	1.74	1.80	1.74	1.64	1.50	1.33	1.14	0.97	0.85

Source of values as in table 3.

In our previous work, we suggested using the mammogram image for localizing the tumor and estimating its size. In this study, the tissues were placed approximately in the center of the Petri dish. The location of the tissue of interest is important information used by the classifiers. This information can be provided by X-rays for the classification of tissues and can be obtained visually when tissue samples are placed in a test system, such as in a Petri dish in vitro.

Obviously the technique developed here for tissue characterization with classifiers does not require cellular phone technology. If a sufficiently powerful computer and data base are available at the patient site the technology can be made self sufficient. However, the use of the cellular phone provides here, as with medical imaging, other advantages. The use of cellular phone technology with classifiers is simple and straightforward. This is particularly important in applications designated for developing areas, where the personnel might be less trained and technical support less available. Another advantage of using electrical impedance measurements is their relatively low price and robust implementation. We believe that the separation of the classifier location from the measurement location can help in providing an updated and accurate classifier while benefiting cost, ease and simplicity of use. A more complete system should receive the new measured data from the remote site and only later (after a period of several months) receive the ground truth results. This can provide a good follow-up mechanism and help in updating and improving the classifier.

### Conclusion

In this study, we continued working toward the two goals presented in our previous studies: 1) developing a non-invasive method for tissue classification; and 2) using cellular technology in order to make these methods more accessible in developing countries. The first goal was examined by an ex-vivo biological model. The results agree with the simulations presented in our previous study and demonstrate with experimental data that using electrical impedance measurements and machine learning methods, such as SVMs, can facilitate good tissue characterization in a non-invasive way. We have also shown that the combination of electrical impedance measurements with cellular phone technology is feasible and can provide a viable and inexpensive alternative to tissue classification and biopsy analysis in areas of the world that currently have limited access to these types of technologies.

## Supporting Information

Appendix S1 A SVM intuitive explanation
(0.19 MB PDF)
