# Supplementary material for: Cellular Phone Enabled Non-Invasive Tissue Classifier
Source: PLoS One. 2009 Apr 13;4(4):e5178. doi: 10.1371/journal.pone.0005178 (PMC2664897; doi:10.1371/journal.pone.0005178)
Supplement: Appendix S1 — A SVM intuitive explanation (0.19 MB PDF) [file pone.0005178.s001.pdf]

Appendix S1

Eleven different frequencies were used in the present study. This means that the dimension of the input vector *x* was 11. Since drawing in this high dimension is not possible, we will assume that we measured by using only two frequencies. This means that our vectors are size 2 and can be drawn on the XY plane. Later, we will generalize to 11 frequencies. When dealing in two dimensions, we are actually looking for a separating line; in three dimensions, we are looking for a separating plane; and above that, we are looking for a hyperplane. Since they can all be described using equation (2), we will use the word hyperplane even in the case of two dimensions.

Figure 6 illustrates the voltage amplitude measurements for two frequencies of two classes. The red dots represent hearts, while the blue stars represent kidneys. Our goal is to separate these two classes using a hyperplane. As shown in the figure, there are many different hyperplanes that can separate these two classes.

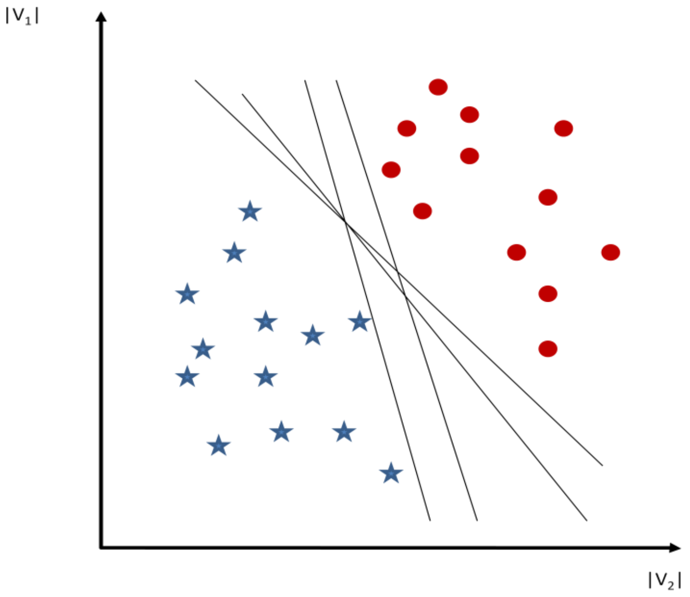

Figure 6 Binary classification example .The red dots represent hearts, and the blue stars represent kidneys. Different separating hyperplanes are drawn.

When using SVMs, we would like to use the hyperplane that maximizes the distance from the closest vector of each class, as seen in Figure 7. These vectors are called “support

vectors.” Equation (2) uses these vectors to define our hyperplane. The assumption made when choosing the hyperplane that maximizes the distance is that any new examples obtained from one of the classes will most likely be classified properly since the largest boundaries possible were drawn.

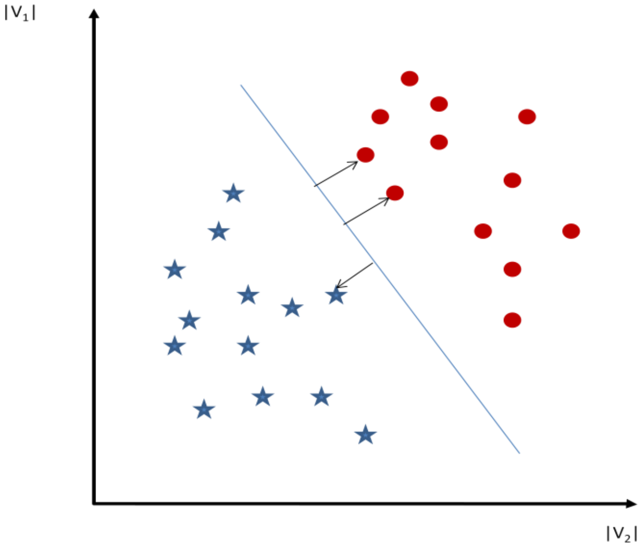

Figure 7: SVM separation example. The two red dots and the one blue star (indicated by the arrows) represent the support vectors. These are the vectors closest to the hyperplane and are used to define it. The arrows represent the distance from the support vectors to the hyperplane and are maximized when choosing the optimal hyperplane.

Since a separating hyperplane may not exist, as can be seen for example in Figure 8, we will not be able to satisfy equation (3). By relaxing the constraints, some misclassification is allowed, leading to equation (9).

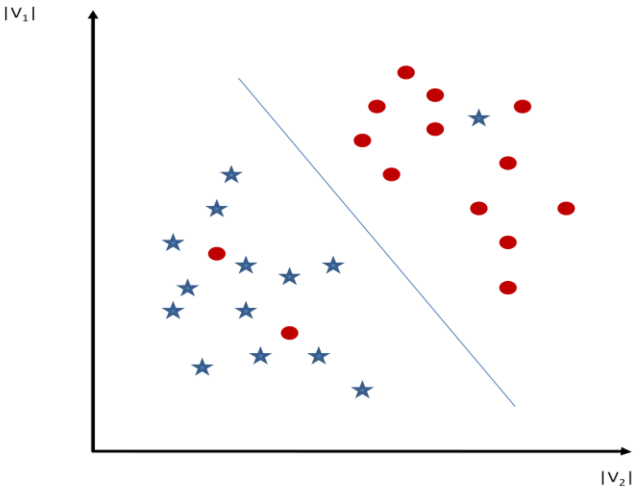

Figure 8: Outliers -An example of a case where there is no hyperplane that can get a perfect separation.

It should be noted that there are some cases in which the data cannot be separated by a hyperplane. In that case, we will use the function φ to map the input space to a feature space where a separating hyperplane might be found. Figure 9 illustrates an example in which a separating hyperplane cannot be found in the Cartesian coordinates, whereas finding this hyperplane is possible if we map the points to Polar coordinates. By using a kernel function, it is possible to compute the separating hyperplane without explicitly mapping the input space to the feature space. For a more complete explanation on kernels, the reader is referred to the introductory chapter of [25].

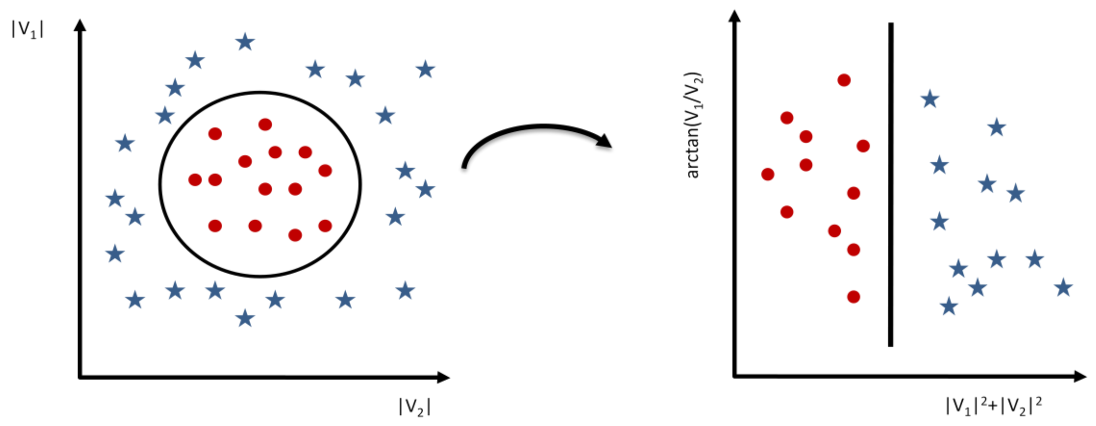

Figure 9: Mapping of feature space. a) Cartesian coordinates; and b) Polar coordinates (the exact transformation would also shift the coordinates). It can be seen that there is no separating hyperplane in the Cartesian coordinates, while it is easily found in the Polar coordinates.
